# Beta Blocker Therapy for Congenital Hepatic Arteriovenous Fistula in Two Neonates

**DOI:** 10.3389/fped.2020.00163

**Published:** 2020-04-21

**Authors:** Hongjun Ba, Lingling Xu, Huimin Peng, Yuese Lin, Xuandi Li, Youzhen Qin, Huishen Wang

**Affiliations:** ^1^Department of Pediatric Cardiology, Heart Center, The First Affiliated Hospital, Sun Yat-sen University, Guangzhou, China; ^2^Department of Pediatrics, The First Affiliated Hospital, Sun Yat-sen University, Guangzhou, China

**Keywords:** hepatic arteriovenous fistula, propranolol, metoprolol, pulmonary arterial hypertension, neonates

## Abstract

**Introduction:** Hepatic arteriovenous fistula (HAVF) is an abnormal communication between the hepatic arteries and hepatic veins. This condition is treated mainly using interventional closure and surgery. However, these procedures are associated with many postoperative complications and high mortality. Propranolol and other beta blockers have been used widely and effectively to treat infantile hemangiomas. However, no reports describe the use of these drugs to treat congenital HAVF.

**Case Description:**Here, we present two cases in which beta blocker therapy was used to treat congenital HAVF in neonates. In both cases, antenatal examinations revealed cardiac enlargement and hepatic space-occupying lesions. After birth, both patients rapidly presented with respiratory distress, cyanosis, and heart failure. Echocardiography suggested enlargement of the right heart, widening of the pulmonary artery, and severe pulmonary arterial hypertension, and hepatic examinations revealed HAVF.

**Results:**After admission, the patients were treated with dopamine, milinone, and furosemide for heart failure. However, their conditions worsened, as indicated by nod-like breathing and cyanosis. Endotracheal intubation and ventilator-assisted breathing and a small dose of oral propranolol (1 mg/kg/d) were initiated. The patients' conditions improved, as indicated by decreases in levels of the N-terminal pro-hormone BNP, and the ventilators were removed. The propranolol dose was increased gradually to 2 mg/kg/d. After 2 weeks of propranolol treatment, the neonate in case 2 developed bronchospasm, which improved after propranolol treatment ended and metoprolol treatment was initiated. Liver imaging performed 8–9 months after beta blocker therapy suggested the disappearance of the arteriovenous fistulae in case 2, and close to disappearing of the arteriovenous fistulae in case 1.

**Conclusion:**Propranolol and metoprolol can effectively treat HAVF in infants, an observation consistent with that found in earlier studies that have shown beta blockers are a valid medical treatment option for infantile hemangioma. However, future studies should explore the underlying potential mechanism.

## Introduction

Hepatic arteriovenous fistula (HAVF) is an abnormal communication between the hepatic arteries and hepatic veins. In adults, liver biopsy, liver tumor, liver surgery, and trauma are common secondary causes of HAVF, while congenital hepatic arteriovenous malformations presents more often in children than in adults (1). The main clinical manifestations of HAVF are severe heart failure and pulmonary hypertension. Congenital HAVF is rare, and children who present with this condition tend to have a more severe condition, more rapid progression, and higher mortality than those with idiopathic disease. Currently, interventional closure and surgery are the main treatment methods for HAVF. However, these procedures are associated with many postoperative complications and high mortality (2, 3), and present serious challenges to clinicians. Propranolol and other beta blockers have been used widely and effectively to treat infantile hemangiomas (4, 5). However, no reports have described the use of these drugs to treat congenital HAVF. In this report, we share our experiences of using beta blockers to treat congenital HAVF in two children, as well as a literature review.

## Case Description

### Case 1

**Figure 1:**
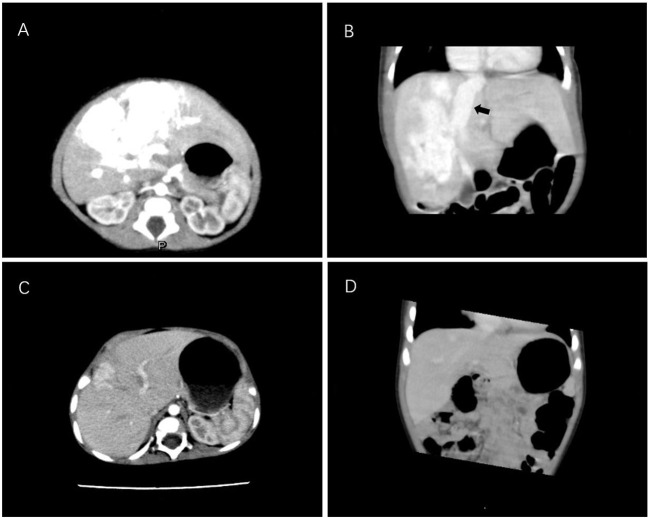
Enhanced CT scan of the liver in case 1. **(A,B)** CT images of the infant at 8 days old reveal a large arteriovenous fistula in the right hepatic lobe. The black arrow indicates the dilated hepatic vein. **(C,D)** A repeated CT scan after 9 months of propranolol treatment reveals that the hepatic arteriovenous fistula was significantly reduced. CT, computed tomography.

A 4-day-old male infant was admitted to our hospital with a 2-day history of respiratory distress. An antenatal examination had observed fetal heart enlargement and an intrahepatic space-occupying lesion. A physical examination upon admission indicated a weight of 3.8 kg, percutaneous oxygen saturation (SPO_2_) of 94%, respiratory rate of 44–60 breaths/min with nasal flaring and retractions, heart rate of 136–160 beats/min, P2 enhancement, and strong heart sounds. No subcutaneous capillary dilatation was observed, and he had no family history of telangiectasia or similar presentation. Echocardiography suggested enlargement of the right heart, widening of the pulmonary artery, mitral valve insufficiency (mild), tricuspid insufficiency (moderate to severe), PFO, and severe pulmonary arterial hypertension (PASP = 55 mmHg). Chest radiographs suggested an increased cardiac shadow and cardiothoracic ratio (0.65). Moreover, his N-terminal pro-hormone BNP (NT-proBNP) level was abnormally high (35,616 pg/ml; normal reference range: 0–84 pg/ml). An enhanced computed tomography (CT) scan of the liver revealed a large arteriovenous fistula in the right hepatic lobe (Figures 1A,B).

After admission, digitalis and diuretics were administered to treat heart failure. On day 4 after admission, the patient presented with respiratory distress (64 breaths/min) and cyanosis (SPO_2_ = 92%) and progressive enlargement of the liver. Dopamine and milinone were administered but failed to improve cardiac function. Mechanical ventilation was applied because his blood oxygen remained low under high-flow oxygen inhalation. The patient was administered a small oral dosage of propranolol (1 mg/kg/d), and his condition improved thereafter. A reexamination revealed that his NT-proBNP level had decreased, and he was successfully weaned off mechanical ventilation after 6 days. The propranolol dosage was increased gradually to 2 mg/kg/d. At 3 months, CT and color Doppler ultrasound analyses of the liver suggested a reduction of the arteriovenous fistula. At 9 months, CT indicated that the arteriovenous fistula had significantly reduced (Figures 1C,D).

### Case 2

**Figure 2:**
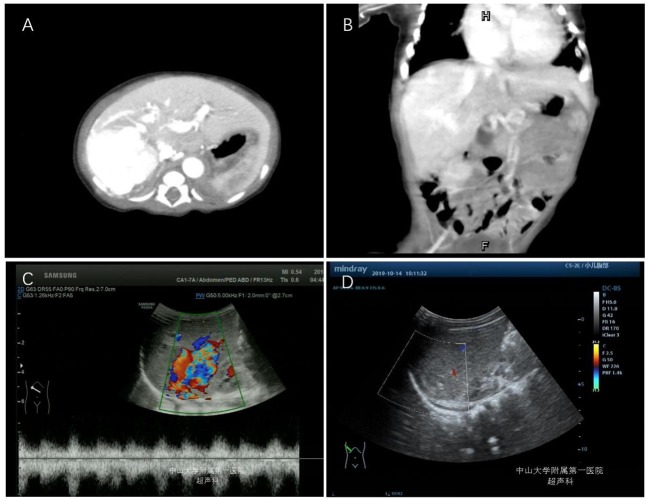
Enhanced CT scan and color Doppler examinations of the liver in case 2. **(A,B)** CT images of the infant at 3 days old. **(C)** Color Doppler analysis of the liver at 3 months after metoprolol treatment. **(D)** Color Doppler analysis of liver at 8 months after metoprolol treatment.

A 2-h-old male infant was admitted to our hospital with dyspnea. An antenatal ultrasound had revealed an enlarged fetal heart and a mixed mass in the liver (dimensions: 53 × 42 × 47 mm). An examination upon admission revealed obvious hyperventilation with a respiratory rate of 80–90 breaths/min, a positive tripitate sign, SPO_2_ of 91–95%, a regular heart rate of 145–165 beats/min, strong heart sounds, a hyperactive second pulmonary artery tone, and hepatomegaly. No subcutaneous capillary dilatation was observed. Echocardiography revealed an enlarged right heart, tricuspid regurgitation, and pulmonary arterial hypertension (PASP = 50 mmHg). An X-ray examination suggested an increased heart shadow. His NT-proBNP level was highly elevated (44,672 pg/ml). An enhanced CT scan of the liver revealed a large arteriovenous fistula in the right hepatic lobe (Figures 2A,B).

After admission, dopamine, milinone, and furosemide were administered for heart failure, and sildenafil was administered for pulmonary hypertension. On the fourth day of admission, the patient's condition worsened, as indicated by nod-like breathing and cyanosis. Mechanical ventilation was initiated. After 7 days, he was successfully weaned off mechanical ventilation, and oral propranolol treatment was initiated at a dosage of 1 mg/kg/d. The patient's blood glucose concentration, heart rate, and blood pressure remained normal. After 2 weeks of propranolol treatment, the patient developed bronchospasms, which improved after the propranolol treatment was discontinued and metoprolol treatment was initiated. The initial dose of metoprolol was 0.5 mg/kg/d and gradually increased to 1.5 mg/kg/d after 2 weeks. At 3 months of age, a repeated color Doppler ultrasound analysis of the liver suggested that the arteriovenous fistula had decreased in size (Figure 2C). The patient's NT-proBNP level was normal. At 8 months of age, color Doppler ultrasound of the liver suggested that the arteriovenous fistula had disappeared (Figure 2D).

## Discussion

In this report, we have described the diagnosis, treatment, and outcomes of two infant patients with congenital HAVF. Most notably, we are the first to report the successful use of beta blockers to treat this condition. This study was approved by the Institutional Review Board of the First Affiliated Hospital of Sun Yat-sen University.

HAVF can be described as a vascular malformation that directly connects the hepatic artery with a portal vein or hepatic vein without passing through the hepatic sinus. This vascular malformation occurs most frequently as hepatic artery portal vein fistula (HAPF) and HAVF, while hepatic porto systemic venous fistula (HPVF) is a less common variant. The main clinical manifestations of HAPF include portal hypertension caused by the direct high-pressure flow of hepatic arterial blood into the portal vein, splenomegaly, esophageal varices and bleeding, and abdominal pain and ascites (6, 7). HAVF, on the other hand, is mainly caused by the direct flow of hepatic arterial blood into the hepatic vein via abnormal channels. This blood then returns to the heart via the inferior vena cava, leading to the aggravation of the pre-cardiac load. The early signs of HAVF include compensable heart enlargement, increased cardiac output and arrhythmia, and late-onset heart failure (8).

HAVF can be classified etiologically as either idiopathic or secondary. The majority of cases are due to a second etiology, the most common of which are iatrogenic injury and liver trauma (e.g., percutaneous liver biopsy, liver intervention, and blunt abdominal injury). In adults, HAVF is a common and characteristic manifestation of liver cancer (9, 10). In children, however, congenital HAVF is most common. Most cases of HAVF are detected during the fetal period, and as neonates, these children present with severe heart failure and pulmonary arterial hypertension (3). As noted in the Introduction, the treatments for HAVF are invasive, associated with complications, and have a high mortality rate; additionally, their efficacy in the treatment of congenital HAVF remains uncertain (11, 12).

The pathogenesis of HAVF is similar to that of infantile hemangioma. Propranolol, metoprolol, and other beta blockers have been used successfully to treat infantile hemangiomas (4, 5). However, the exact mechanism by which propranolol can treat infantile hemangioma is not fully understood. In one study, propranolol appeared to repress endothelial cell growth and promote apoptosis in infantile hemangioma by upregulating the expression of microRNA (miR)-125b, which in turn suppressed tumor development by targeting TFAP4 (13). Moreover, propranolol was shown to promote apoptosis in hemangiomas by downregulating the expression of miR-4295 (14). Based on the efficacy of propranolol in the treatment of infantile hemangioma, we administered propranolol to our two infant patients with severe HAVF. Our patients experienced gradual improvements in heart failure and pulmonary hypertension, as well as gradual decreases in the sizes of the fistulae, suggesting that propranolol could also effectively treat congenital HAVF in infants. During treatment, both patients' blood glucose concentrations, blood pressure levels, and heart rates remained within normal ranges, and no notable adverse reactions occurred.

The main reported side effects of propranolol are hypoglycemia and induced bronchospasms. Therefore, clinicians should pay attention to these adverse reactions during the treatment process. In the second case, the patient developed asthma and required a change to metoprolol, which is highly selective and less likely to cause asthma; this drug was well-tolerated. Currently, the mechanisms by which propranolol and metoprolol can effectively treat HAVF remain unclear, and further study is needed. Possibly, the mechanism may be similar to the effects observed when propranolol is used to treat infantile hemangioma, described above.

In conclusion, our observations suggest that propranolol and metoprolol can effectively treat HAVF in infants, a finding that is consistent with previous reports of the effective use of propranolol for the treatment of infantile hemangioma. However, very few cases have been observed to date, and the sample size needs to be expanded in future studies.

## Data Availability Statement

The raw data supporting the conclusions of this article will be made available by the authors, without undue reservation, to any qualified researcher.

## Ethics Statement

Written informed consent for the publication of this case report was obtained from the parents of the patients.

## Author Contributions

All authors listed have made a substantial, direct and intellectual contribution to the work, and approved it for publication.

## Conflict of Interest

The authors declare that the research was conducted in the absence of any commercial or financial relationships that could be construed as a potential conflict of interest.
